# A Nested PCR Assay to Avoid False Positive Detection of the Microsporidian *Enterocytozoon hepatopenaei* (EHP) in Environmental Samples in Shrimp Farms

**DOI:** 10.1371/journal.pone.0166320

**Published:** 2016-11-10

**Authors:** Pattana Jaroenlak, Piyachat Sanguanrut, Bryony A. P. Williams, Grant D. Stentiford, Timothy W. Flegel, Kallaya Sritunyalucksana, Ornchuma Itsathitphaisarn

**Affiliations:** 1 Department of Biochemistry, Faculty of Science, Mahidol University, Bangkok, Thailand; 2 Center of Excellence for Shrimp Molecular Biology and Biotechnology (Centex Shrimp), Faculty of Science, Mahidol University, Bangkok, Thailand; 3 Shrimp Pathogen Interaction Laboratory (SPI), National Center for Genetic Engineering and Biotechnology (BIOTEC), Bangkok, Thailand; 4 Biosciences, College of Life and Environmental Sciences, University of Exeter, Exeter, United Kingdom; 5 European Community Reference Laboratory for Crustacean Diseases, Center for Environment, Fisheries and Aquaculture Science (Cefas), Weymouth, Dorset, United Kingdom; 6 National Center for Genetic Engineering and Biotechnology (BIOTEC), National Science and Technology Development Agency (NSTDA), Pathum Thani, Thailand; Institute of Plant Physiology and Ecology Shanghai Institutes for Biological Sciences, CHINA

## Abstract

Hepatopancreatic microsporidiosis (HPM) caused by *Enterocytozoon hepatopenaei* (EHP) is an important disease of cultivated shrimp. Heavy infections may lead to retarded growth and unprofitable harvests. Existing PCR detection methods target the EHP small subunit ribosomal RNA (SSU rRNA) gene (SSU-PCR). However, we discovered that they can give false positive test results due to cross reactivity of the SSU-PCR primers with DNA from closely related microsporidia that infect other aquatic organisms. This is problematic for investigating and monitoring EHP infection pathways. To overcome this problem, a sensitive and specific nested PCR method was developed for detection of the spore wall protein (SWP) gene of EHP (SWP-PCR). The new SWP-PCR method did not produce false positive results from closely related microsporidia. The first PCR step of the SWP-PCR method was 100 times (10^4^ plasmid copies per reaction vial) more sensitive than that of the existing SSU-PCR method (10^6^ copies) but sensitivity was equal for both in the nested step (10 copies). Since the hepatopancreas of cultivated shrimp is not currently known to be infected with microsporidia other than EHP, the SSU-PCR methods are still valid for analyzing hepatopancreatic samples despite the lower sensitivity than the SWP-PCR method. However, due to its greater specificity and sensitivity, we recommend that the SWP-PCR method be used to screen for EHP in feces, feed and environmental samples for potential EHP carriers.

## Introduction

Microsporidia are obligate, intracellular, spore-forming parasites [1]. The spores of microsporidia have a double-layered chitinaceous wall embedded with proteins that are believed to be involved in host cell invasion and tissue recognition [2–4]. They infect a wide range of host animal species from invertebrates to vertebrates, with infections ranging from sub-lethal to lethal effects depending on pathogen load and host condition [5]. Microsporidian infections by *Nosema ceranae* and *N*. *apis* in honeybees can lead to increased mortality and colony collapse [6], and infection by *Enterocytozoon bieneusi* in immunocompromized humans may cause severe diarrhea and death [7].

Aquatic animals, including freshwater fish, marine lobsters, crabs, copepods and shrimp have been found to be infected with various genera of Microsporidia [8–14]. As one of the world’s largest shrimp producers [15], Thailand’s shrimp production has been negatively impacted by two microsporidia, namely *Agmasoma penaei* and *Enterocytozoon hepatopenaei* (EHP). *A*. *penaei* was first discovered in Thailand in 1992 and caused ‘cotton shrimp’ disease or ‘white back’ disease [16]. However, as *A*. *penaei* cannot be horizontally transmitted among shrimp, the negative impact of this microsporidian species was ameliorated by removing the suspected fish alternate hosts from the shrimp cultivation system [17]. In contrast, EHP differs markedly from *A*. *penaei* in that infections can be spread horizontally in shrimp ponds by cannibalism [18] and cohabitation [19] making it a much more serious threat to shrimp farmers.

EHP is the causative agent of hepatopancreatic microsporidiosis (HPM) and was first recognized as an unidentified microsporidian in the tubule epithelial cells of the hepatopancreas in *Penaeus monodon* in Thailand in 2004 [20]. It was later characterized [12] and subsequently found in the more economically important *P*. *vannamei* [18]. Unpublished reports from farmers suggest that EHP is involved in the retarded growth of shrimp. This is consistent with a recent report from China that showed a negative correlation between shrimp size and EHP load above 10^3^ copies/ng of total shrimp DNA [21].

For control of hepatopancreatic microsporidiosis in shrimp, a major initial focus was to exclude EHP-infected broodstock and their post larvae from the cultivation system. This has been accomplished in part by screening broodstock, post larvae, and living feed, such as brine shrimp (*Artemia*), for post larvae, and molluscs or polychaetes for broodstock with nested PCR that targets the small subunit ribosomal RNA (SSU rRNA) gene of EHP (SSU-PCR) [18]

However, the level of threat from environmental sources of infection in rearing ponds is still unknown. Issues of concern are the viability of residual spores that may be present in previously infected ponds and the existence of infected carrier species that may comprise an environmental reservoir. Positive SSU-PCR test results for molluscs and polychaetes should be followed up by *in situ* hybridization assays to determine whether they are active (infected) or passive (uninfected) carriers. In addition, after the development of the SSU-PCR method [18], we discovered that recently published SSU rRNA sequences of closely related microsporidia in marine organisms may potentially give false positive test results for EHP. As hosts of some of these microsporidia, for example fish and *Artemia*, are raw materials of shrimp feed, such false positive test results from the SSU-PCR methods might lead to unnecessary destruction of feed and broodstock from which feces are used for non-invasive PCR diagnosis.

All of the current molecular tools for EHP detection via nucleic acids are based on targeting the SSU rRNA gene. They include conventional PCR, nested PCR, isothermal loop-mediated amplification (LAMP) and *in situ* hybridization [18,22,23]. Thus, to explore the real possibility of obtaining false positive test results by using the SSU-PCR methods, we tested DNA extracted from microsporidia that are closely related to EHP and available to us. We demonstrated that, while the SSU-PCR method [18] produced false positive test results with DNA from closely related microsporidia, a newly developed nested PCR method based on a spore wall protein (SWP) gene (SWP-PCR) of EHP was more sensitive than the SSU-PCR in the first PCR reaction and more discriminatory overall.

## Materials and Methods

### 1. Multiple sequence alignment analysis

The SWP gene sequence for EHP used in this work was obtained by whole genome sequencing of DNA extracted from EHP spores purified from infected hepatopancreatic tissue by Percoll gradient centrifugation. It has been submitted to the GenBank database and assigned the accession number KX258197. Accession numbers of the nucleotide sequences of the SSU rRNA and SWP genes from EHP-related microsporidian taxa were retrieved from the GenBank database and are shown in Tables 1 and 2, respectively. Multiple sequence alignments were carried out using Clustal Omega (http://www.ebi.ac.uk/Tools/msa/clustalo) [24].

**Table 1:** Small subunit rRNA sequences used for multiple sequence alignment analysis.

Microsporidian species	Acronym	%Identity	Host species	Accession No.
*Enterocytozoon hepatopenaei*	EHP	-	*Penaeid* spp.	KP759285.1
*Enterospora nucleophile*	*Enu*	93	*Sparus aurata*	KF135645.1
*Enterospora canceri*	*Eca*	90	*Cancer pagurus*	HE584634
*Nucleospora salmonis*	*Nsa*	89	Salmonidae	AF185991.1
*Enterocytozoon bieneusi*	*Ebi*	88	*Homo sapiens*	AY257180.1
*Nucleospora cyclopteri*	*Ncy*	86	*Cyclopterus lumpus*	KC203457.1
*Obruspora papernae*	*Opa*	86	*Callionymus filamentosus*	HG005137.1
*Paranucleospora theridion*	*Pth*	86	*Salmo salar* and *Lepeophtheirus salmonis*	FJ594988.1
*Enterocytospora artemiae*	*Ear*	82	*Artemia* spp.	JX839889.1
*Hepatospora eriocheir*	*Her*	80	*Eriocheir sinensis*	HE584635.1

**Table 2:** Spore wall protein sequences used for multiple sequence alignment analysis.

Microsporidian species	Acronym	% Identity	Host species	Accession No.
*Enterocytozoon hepatopenaei*	EHP	-	*Penaeid* spp.	KX258197
*Enterocytozoon bieneusi*	*Ebi*	66	*Homo sapiens*	NW003102063.1 (38817–39503)
*Enterospora canceri*	*Eca*	64	*Cancer pagurus*	Unpublished
*Hepatospora eriocheir*	*Her*	60	*Eriocheir sinensis*	Unpublished

### 2. Shrimp specimens

With permission from the farm owners to collect specimen from their properties for this study, EHP-infected *P*. *vannamei* (5–7 grams) were collected from commercial shrimp ponds in Trat province, Thailand from August to September 2015. From each shrimp, hepatopancreas was removed, being careful to exclude bacterial contamination from the stomach and intestine. One half of the hepatopancreas was subjected to DNA extraction while the other was preserved with Davidson’s fixative and processed for routine paraffin embedding and histological analysis as described by Bell & Lightner [25].

### 3. DNA extraction and purification

Hepatopancreatic tissue was homogenized in 500 μl lysis buffer (50 mM Tris pH 9, 0.1 M EDTA pH 8, 50 mM NaCl, 2% SDS) containing 5 μg/ml proteinase K before incubation at 55°C for 30 min. Total DNA was purified using a standard phenol-chloroform method [26] and treated with DNase-free RNase (New England Biolabs, USA). Concentration of DNA was determined using a NanoDrop Spectrophotometer (Thermo Scientific, USA).

### 4. SSU-PCR and SWP-PCR detection methods

The nested SSU rRNA PCR method (SSU-PCR) used in this study has been previously described [18] and the primers for it are shown in Table 3. For the nested SWP PCR method (SWP-PCR), primers were designed from the SWP sequence of EHP (GenBank Accession no. KX258197) using Primer3 software [27]. Secondary structures of the primers were analyzed using the Mfold web server [28]. The PCR reaction mixture for both steps (25 μl) contained 0.2 mM dNTP, 1.5 mM MgCl_2_, 0.2 μM of each primer, 0.5 unit of *Taq* DNA polymerase (New England Biolabs, USA). For the first step PCR, added templates consisted of either 100 ng of total DNA extracted from EHP-infected, shrimp hepatopancreatic tissue or 5 ng of control plasmid pGEM-SWP (see below). The PCR protocol for the first PCR reaction used primers SWP_1F and SWP_1R (Table 3) and consisted of a 5-min initial denaturation at 95°C followed by 30 cycles of denaturation for 30 s at 95°C, annealing for 30 s at 58°C and extension for 45 s at 68°C with a final 5-min extension step at 68°C. The expected PCR product was 514 bp. For the second (nested) PCR step, the template consisted of 1 μl of the final reaction solution from the first PCR step. The PCR protocol for the second, nested PCR reaction used primers SWP_2F and SWP_2R (Table 3), with an initial denaturation at 95°C for 5 min followed by 20 cycles of 30 s denaturation at 95°C, 30 s annealing at 64°C and 20 s extension at 68°C with a final extension for 5-min at 68°C. The expected PCR product was 148 bp. The amplicons were analyzed by 1.5% agarose gel electrophoresis with ethidium bromide staining and using a DNA ladder marker (2 log, 100 bp, or 1 kb DNA ladder from New England Biolabs, USA)

**Table 3:** PCR primers used in this study.

Primer name	Sequence (5’ to 3’)	Amplicon size (bp)	Reference
**SWP-PCR**			
**First step**			
SWP_1F	TTGCAGAGTGTTGTTAAGGGTTT	514	This study
SWP_1R	CACGATGTGTCTTTGCAATTTTC		
**Nested step**			
SWP_2F	TTGGCGGCACAATTCTCAAACA	148	This study
SWP_2R	GCTGTTTGTCTCCAACTGTATTTGA		
**SSU-PCR**			
**First step**			
ENF779	CAGCAGGCGCGAAAATTGTCCA	779	[18]
ENR779	AAGAGATATTGTATTGCGCTTGCTG		
**Nested step**			
ENF176	CAACGCGGGAAAACTTACCA	176	[18]
ENR176	ACCTGTTATTGCCTTCTCCCTCC		
**Actin PCR**			
Actin_F	CCTCGCTGGAGAAGTCCTAC	401	[29]
Actin_R	TGGTCCAGACTCGTCGTACTC		

### 5. Construction of a plasmid control template for SWP-PCR

To construct a plasmid containing a fragment of the SWP gene, the primers SWP_1F and SWP_1R (Table 3) were used as described above to generate a 514 bp amplicon that was cloned into a pGEM®-T Easy Vector (Promega, USA). Plasmids from positive transformants were extracted using a Presto^TM^ Mini Plasmid kit (Geneaid, Taiwan) and sequenced using both SP6 and T7 universal primers (Macrogen, South Korea). Nucleotide sequences were analyzed by BLASTn (http://www.ncbi.nlm.nih.gov/BLAST) and aligned against the SWP sequence (GenBank Accession no. KX258197) using MUSCLE multiple sequence alignment software (http://www.ebi.ac.uk/Tools/msa/muscle). The plasmid was named pGEM-SWP. This plasmid and one (pGEM-SSU) containing the target for the SSU-PCR method [18] were used as positive control templates and for testing the comparative sensitivity of the SWP-PCR and SSU-PCR detection methods.

### 6. Specificity of the SWP-PCR and SSU-PCR detection methods

To test the specificity of the SWP-PCR and SSU-PCR methods, PCR reactions were carried out as described in section 4, with the exception that the total reaction volume contained 20 ng of total DNA (gDNA) extracted from aquatic organisms infected with other microsporidian species. These were closely-related *Enterospora canceri (Eca)* from the European edible crab (*Cancer pagurus*) and *Hepatospora eriocheir (Her)* from the Chinese mitten crab, which were chosen for the specificity test because of their availability in our laboratory. The more distantly related microsporidia are *Thelohania sp*. *(The)* from white clawed crayfish and *Spraguea lophii (Slo)* from the monkfish *Lophius piscatorius* and *Lophius budegassa*. Positive control reactions included the plasmids (+ve; pGEM-SSU and pGEM-SWP plasmids for their respective primers) and total hepatopancreatic DNA from EHP-infected shrimp (I). Negative control reactions included total hepatopancreatic DNA from naïve shrimp (U) and water (-ve). The PCR conditions were performed and analyzed as described above.

### 7. Comparative sensitivity of the SWP-PCR and SSU-PCR methods

To compare the sensitivity of the SSU-PCR and SWP-PCR methods, plasmids pGEM-SWP and pGEM-SSU were used as serially diluted templates for their corresponding PCR reactions. The highest dilution that still gave a visible band on the agarose gel was considered the lowest detectable quantity of target DNA and the equivalent copy number was calculated using Avogadro’s number against the molar quantity of plasmid DNA.

### 8. Comparison of SSU-PCR and SWP-PCR with field samples

With permission from the farm owners to collect specimen from their properties for this study, we used a total of 25 DNA extracts from hepatopancreatic tissue of EHP-infected shrimp that had previously been obtained from commercial shrimp farms. These specimen had previously given positive PCR test results with the SSU-PCR method [18] and exhibited histological evidence of EHP infection. The DNA were subjected to a second round of testing using both the SSU-PCR [18] and SWP-PCR methods to test the consistency of the two methods. For an internal control PCR reaction, primers for a *P*. *vannamei* actin gene [29] (Table 3) were used in a 25-μl reaction which contained 100 ng total shrimp DNA, 0.2 mM dNTP, 1.5 mM MgCl_2_, 0.2 μM of each primer, 0.5 unit of *Taq* DNA polymerase (New England Biolabs, USA). The condition for the actin PCR reaction was 5-min initial denaturation at 95°C followed by 30 cycles of denaturation for 30 s at 95°C, annealing for 30 s at 55°C and extension for 45 s at 68°C with a final 5-min extension step at 68°C. The expected PCR amplicon of actin was 401 bp.

### 9. Preparation of a SWP *in situ* hybridization probe

The primers SWP_1F and SWP_1R and the plasmid pGEM-EHP were used to prepare a DIG-labeled SWP probe for *in situ* hybridization assays following the protocol described by Tangprasittipap *et al*. [18] Briefly, a DIG-PCR labeling kit (Roche, Germany) was used and the probe was purified using a PCR-amplicon purification kit (Geneaid, Taiwan), after which labeling efficiency was determined by dot blot hybridization.

The hybridization protocol also followed Tangprasittipap *et al*. [18]. Briefly, tissue sections were treated with TNE buffer containing 5 μg/ml proteinase K and incubated at 37°C for 10 min in a humidified chamber. The sections were then incubated with 0.4% formaldehyde for 5 min, 0.5 M EDTA for 1 hour, and pre-hybridization buffer (4x SSC buffer (3M NaCl, 0.3M sodium citrate) and 50%(v/v) deionized formamide) for 10 min. Approximately 200 ng of the DIG-labeled SSU probe or DIG-labeled SWP probe were mixed with hybridization buffer (4x SSC buffer, 50% deionized formamide, 1x Denhardt’s solution (Sigma, USA), 0.25 mg/ml salmon sperm DNA (Invitrogen, USA), 5% (w/v) dextran sulfate) and overlaid on rehydrated tissue sections followed by incubation at 42°C overnight in a humid chamber. Stringency washes were carried out using SSC buffer and buffer I (1M Tris-HCl, 1.5M NaCl) at 37°C before 0.5% blocking solution (Roche, Germany) was added. For detection, the slides were treated with 1:500 alkaline phosphatase-conjugated anti-DIG antibody. Buffer I was used twice to wash away unbound materials. Development of signals was carried out using BCIP/NBT solution (Roche, Germany). Finally, sections were counterstained in 0.5% Bismarck brown Y (Sigma, USA) and washed with running tap water for 10 min before dehydration and mounting for light microscopy.

## Results

### 1. False positive SSU-PCR results for EHP

**Fig 1:**
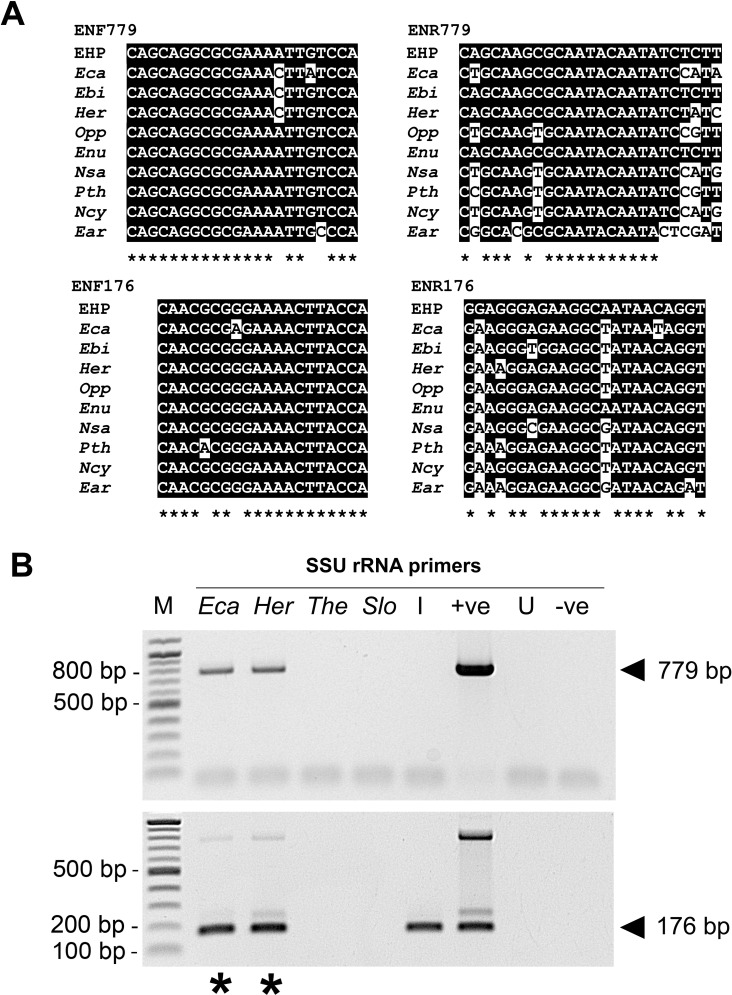
Alignments of the SSU-PCR primer sequences and confirmation of cross reactions with closely related microsporidia. (A) Alignments of the SSU primer sequences (Table 3) with homologous SSU regions of other microsporidia (Table 1). Black highlights indicate matches with the primer sequences, while asterisks under the sequences indicate regions of 100% identity for all of the aligned sequences. (B) Agarose gel of SSU-PCR amplicons from EHP and other microsporidia. In addition to the pGEM-SSU plasmid (+ve) and water (-ve), total DNA obtained from EHP-infected shrimp (I) and naïve shrimp (U) were used as controls. PCR amplicons and false positive test results are marked with arrowheads and asterisks, respectively. The band at 226 bp show amplicons of residual primers ENR779 from the first PCR step and primers ENF176 from the second nested PCR step.

Unpublished reports from producers of shrimp feed indicated that the SSU rRNA primers developed in Tangprasittipap et al., 2013 [18] produced false positive results when used to screen raw materials such as fish meal. To investigate whether the EHP SSU rRNA primers could potentially amplify homologous regions from other closely related microsporidia (Table 1), the SSU rRNA genes of microsporidian pathogens of aquatic hosts, which may contaminate raw materials of shrimp feed, were compiled from the NCBI database. Multiple sequence alignments were carried out and revealed that the homologous regions were highly conserved (Fig 1A). The sequences at the annealing sites of the primers ENF779, ENR779, ENF176 and ENR176 are 86.4%, 66.7%, 90% and 74% identical to that of other microsporidia. This indicated that false positive results might be possible with some EHP-related microsporidia when using the SSU-PCR method.

Primer cross reactivity was tested using SSU-PCR with DNA extracts from aquatic animals infected with microsporidia that are closely related to EHP, namely *Enterospora canceri (Eca)* and *Hepatospora eriocheir (Her)*, or microsporidia that are more distantly related to EHP, namely *Thelohania* sp. *(The)* and *Spraguea lophii (Slo)* (Fig 1B). The negative controls gave negative results, as did total DNA templates containing more distantly related *Thelohania* sp. and *S*. *lophii*. However, the closely related *E*. *canceri* and *H*. *eriocheir* gave false positive results. The sizes of the PCR products from the two crab microsporidia are identical to those obtained with total DNA extracts obtained from EHP-infected shrimp.

### 2. Lack of false positive results for EHP using SWP-PCR

**Fig 2:**
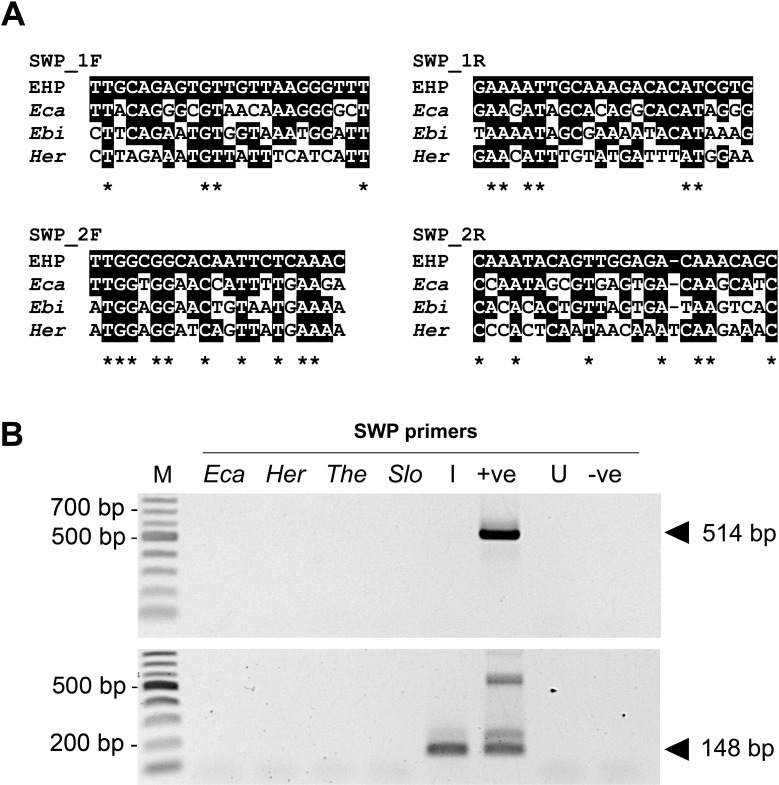
Alignments of the SWP-PCR primer sequences and lack of cross reactions with closely related microsporidia. (A) Alignments of the SWP primer sequences (Table 3) with homologous regions of spore wall protein genes of other microsporidia available in databases (Table 2). Black highlights indicate matches with the primer sequences, and asterisks indicate regions of 100% identity for all of the aligned sequences. (B) Agarose gel of SWP-PCR amplicons from EHP and other microsporidia. In addition to the pGEM-SWP plasmid (+ve) and water (-ve), total DNA obtained from EHP-infected shrimp (I) and naïve shrimp (U) were used as controls. PCR amplicons are marked with arrowheads. The 180 bp band is PCR products from residual primers SWP1_R from the first PCR step and primers SWP_2F from the second nested PCR step.

Due to the false positive results obtained for EHP using the SSU-PCR method, we developed a more discriminatory PCR method using the sequence of the newly discovered, putative spore wall protein (SWP) gene of EHP (GenBank accession number KX258197). An alignment of the designed primers with homologous regions of other SWP sequences (Fig 2A) demonstrates that the degree of sequence similarity among the sequences is lower than that amplified from the SSU rRNA primers (Fig 1A), suggesting that the amplicons from the SWP region would be better at distinguishing EHP from other closely related microsporidia in PCR assays.

Subsequent tests similar to those carried out using SSU-PCR (Fig 1B) were repeated using the SWP-PCR method with the same DNA templates. Only the positive control plasmid DNA and the DNA extracted from EHP-infected shrimp gave positive test results (Fig 2B).

### 3. Comparative sensitivity of the SWP-PCR and SSU-PCR methods

**Fig 3:**
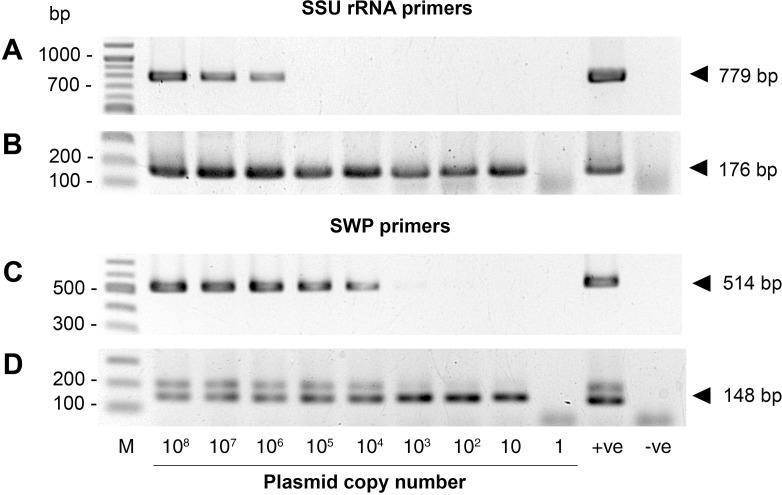
Higher sensitivity of first step SWP-PCR compared to first step SSU-PCR. (A) and (C) show agarose gels of amplicons from the first step PCR reactions, while (B) and (D) show agarose gels of amplicons from the nested step PCR reactions carried out using serial dilutions of the plasmid templates pGEM-SWP and pGEM-SSU, respectively.

Using serially diluted plasmid DNA as templates, SSU-PCR gave the 779-bp amplicon in the first PCR step at 10^6^ copies of the pGEM-SSU plasmid per reaction mix, while SWP-PCR gave the amplicon of 514 bp at 10^4^ copies (Fig 3A). In the nested PCR step, both methods had identical sensitivity at 10 copies per reaction vial (Fig 3B), similar to what had previously been reported for the SSU-PCR method [23].

At high copy numbers of the target sequence in which the first step PCR amplicon was seen, both detection methods resulted in an additional faint band just above their respective nested PCR amplicons. These additional bands were amplified by residual primers from the first PCR step and primers from the second nested step. Specifically, in the SSU-PCR method, the 226 bp amplicon right above the nested 176 bp amplicon were produced from the forward nested primer ENF176 and the reverse first step primer ENR779, while in the SWP-PCR method the 180 bp amplicon just above the nested 148 bp amplicon arose from an interaction between the forward nested primer SWP_2F and the reverse first step primer SWP_1R.

### 4. Comparison of SSU-PCR and SWP-PCR with field samples

**Fig 4:**
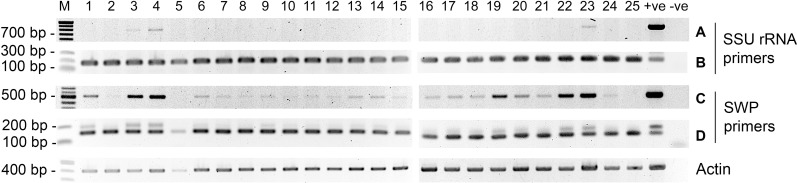
Comparison of the SWP-PCR and SSU-PCR methods with field samples. (A) and (C) are amplicons from the first PCR reactions, while (B) and (D) are amplicons from the nested PCR step. PCR reactions for the housekeeping gene actin were used as the internal control.

Using a total of 25 DNA extracts from hepatopancreas of EHP-infected, farmed shrimp that previously tested positive for EHP using the SSU-PCR method, a second round of tests carried out using both the SSU-PCR and SWP-PCR methods gave positive test results for EHP for all 25 samples with both methods (Fig 4). However, consistent with the greater sensitivity of the first PCR step of the SWP-PCR method (Fig 3), detection of EHP after the first step of PCR was found in 88% of the samples, while only 12% of the samples gave positive results in the first step with the SSU-PCR method.

### 5. SSU and SWP *in situ* hybridization results are similar

**Fig 5:**
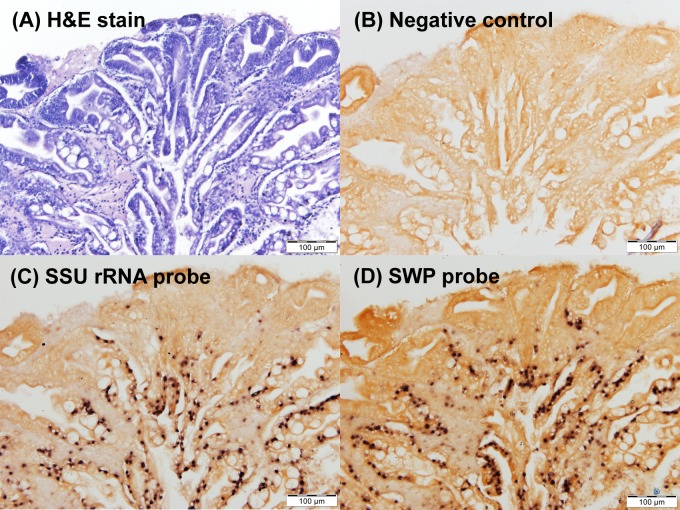
DIG-labeled SWP and SSU probes give comparable *in situ* hybridization results in EHP-infected shrimp. Adjacent hepatopancreatic tissue sections from an EHP-infected shrimp specimen were stained with H&E and tested with the two probes. (A) Section stained with H&E (B) Negative control for *in situ* hybridization (no probe applied) (C) *In situ* hybridization with DIG-labeled SSU rRNA probe (D) *In situ* hybridization with DIG-labeled SWP probe. Black precipitates indicate positive hybridization reactions with EHP.

*In situ* hybridization (ISH) is an important tool to determine the location of pathogen nucleic acid in tissue sections of PCR positive animals in order to know whether they are active (infected) or passive (uninfected) carriers. Blocks of shrimp hepatopancreatic tissue (previously confirmed for EHP infection by SSU-PCR, histology and ISH using the SSU rRNA probe) were used to cut adjacent tissue sections for comparison of *in situ* hybridization reactions using DIG-labeled probes for the SSU rRNA and SWP genes. One tissue section was stained with hematoxylin and eosin (H&E) (Fig 5A) and another served as the negative no-probe control (Fig 5B). The latter showed no positive ISH reaction (black precipitate). However, both the SSU rRNA probe and the SWP probe gave positive ISH reactions for EHP in the same areas of the infected hepatopancreas and at similar intensity (Fig 5C and 5D, respectively). The results showed that either probe could be used for ISH to confirm the location of EHP infected cells in PCR-positive specimens of cultivated *P*. *monodon* and *P*. *vannamei* and other EHP-infected carriers. However, neither of the probes could be used solely to diagnose EHP infections since specificity of the *in situ* hybridization reaction can be relatively low. For example, hepatopancreatic parvovirus (HPV) in *P*. *chinensis* from Korea and *P*. *monodon* from Thailand share approximately 80% nucleic acid identity but an ISH probe based on the HPV sequence from *P*. *chinensis* could sometimes give positive ISH reactions using shrimp infected with HPV from Thailand [30].

## Discussion

In this study, we developed a new, specific, nested PCR method for detection of EHP based on one of the spore wall protein (SWP) genes. Spore walls of microsporidia provide environmental protection and are also involved in host-pathogen interactions [3,31] via species-specific SWP. The SWP-PCR method was superior to the SSU-PCR method in terms of both specificity and sensitivity. Compared to the existing SSU-PCR methods, the new SWP-PCR method did not cross react with DNA from the closely related microsporidia and is more sensitive in the first PCR step.

The low specificity of diagnostic methods based on the SSU rRNA sequence is not limited to EHP. For single-step PCR detection of the malaria parasite *Plasmodium knolesi*, SSU rRNA primers could cross react with *P*. *vivax* and other *Plasmodium* species [32]. Similarly, single-step PCR detection methods for the protozoan parasite *Leishmania siamensis* based on the SSU rRNA gene and a heat shock protein 70 (Hsp70) gene gave false positive results when used with the protozoan parasites *Trypanosoma brucei and T*. *evansi* [33].

Prior to the development of the SWP-PCR method, the SSU-PCR method was used widely to screen for EHP in shrimp, shrimp pond sediments and living, freshly killed or frozen materials used to feed shrimp [34]. Since the SSU-PCR and SWP-PCR methods both gave the same results for all tested specimens of shrimp hepatopancreatic tissue, it suggests that only one microsporidian species is the cause of current HPM outbreaks in cultivated *P*. *monodon* and *P*. *vannamei* in Thailand and elsewhere in Asia. Thus, the SSU-PCR method used here [18] and a more recent one that is also based on the EHP SSU rRNA sequence [22] are still appropriate for use with cultivated shrimp specimens. However, for environmental samples, such as sediments and suspected carriers previously reported to be SSU-PCR positive for EHP infection, it is necessary to re-confirm their status by use of the SWP-PCR method or by sequencing.

The multiple sequence alignments and the PCR test results from the available specimens revealed that false positive test results may occur with the SSU rRNA based methods and that samples of shrimp or shrimp feed might give false positive test results, potentially leading to their unnecessary discard or destruction. In addition, non-destructive screening of broodstock shrimp for EHP in a hatchery is usually carried out using feces and this raises the possibility that PCR positive results might arise from presence of residual microsporidian DNA that originated from the feed source and not from the shrimp themselves. For such reasons, we recommend that non-destructive screening of broodstock feces be carried out using the SWP-PCR method and that any suspected positive broodstock results be confirmed by absence of positive results in their feed.

With respect to sensitivity in testing EHP, the quantitative real-time PCR method [21] may be the most sensitive. However, for those without equipment to carry out the process, an isothermal loop mediated (LAMP) method with a sensitivity of 2 EHP copies/reaction vial of total DNA from EHP-infected shrimp has been reported [23]. In this study, we found that the SSU-PCR and SWP-PCR methods could detect DNA plasmids containing respective target sequences at 10 copies per reaction vial, although the SWP-PCR method had better sensitivity than the SSU-PCR method for the first PCR step.

The greater sensitivity of the SWP primers in the first PCR step might be due to the lower efficiency of the primers and condition for the first step of SSU-PCR. There is a 7°C difference in the melting temperatures of the primers ENF779 and ENR779. The GC content of the SWP primers is also lowered compared to that of the SSU rRNA primers (S1 and S2 Tables). Primers with higher GC contents tend to form more thermodynamically stable secondary structures that can hinder template annealing and therefore, compromise primer efficiency [35–37]. Based on secondary structure analysis of the primers (S1 and S2 Tables), the SSU rRNA primers form secondary structures with more negative, i.e. more thermodynamically favorable, Gibb’s free energy values (ΔG), while the secondary structures formed by the SWP primers have less favorable ΔG values. The enhanced sensitivity is useful for the identification of the potential carriers of EHP in environmental samples. Moreover, the 58°C annealing temperature in the first SSU-PCR step is higher than the 57°C melting temperature of the primer ENR779. Hence, we recommend the new primer sets reported in this study be used as screening primers to study the life cycle of EHP.

In conclusion, we developed a new nested PCR detection method for EHP infection that has superior specificity and sensitivity compared to previous methods. This new method can be used for diagnosis of EHP in shrimp and environmental samples. It will be a useful tool for studying EHP transmission routes with the objective of devising more effective HPM management and control measures.

## Supporting Information

S1 Table GC content and stability of secondary structures of the SWP primers. (DOCX)

S2 Table GC content and stability of secondary structures of the SSU rRNA primers. (DOCX)
