# Supplementary figures and images for: A Nested PCR Assay to Avoid False Positive Detection of the Microsporidian Enterocytozoon hepatopenaei (EHP) in Environmental Samples in Shrimp Farms
Source: PLoS One. 2016 Nov 10;11(11):e0166320. doi: 10.1371/journal.pone.0166320 (PMC5104377; doi:10.1371/journal.pone.0166320)

**S1 Table. GC content and stability of secondary structures of the SWP primers.**

**
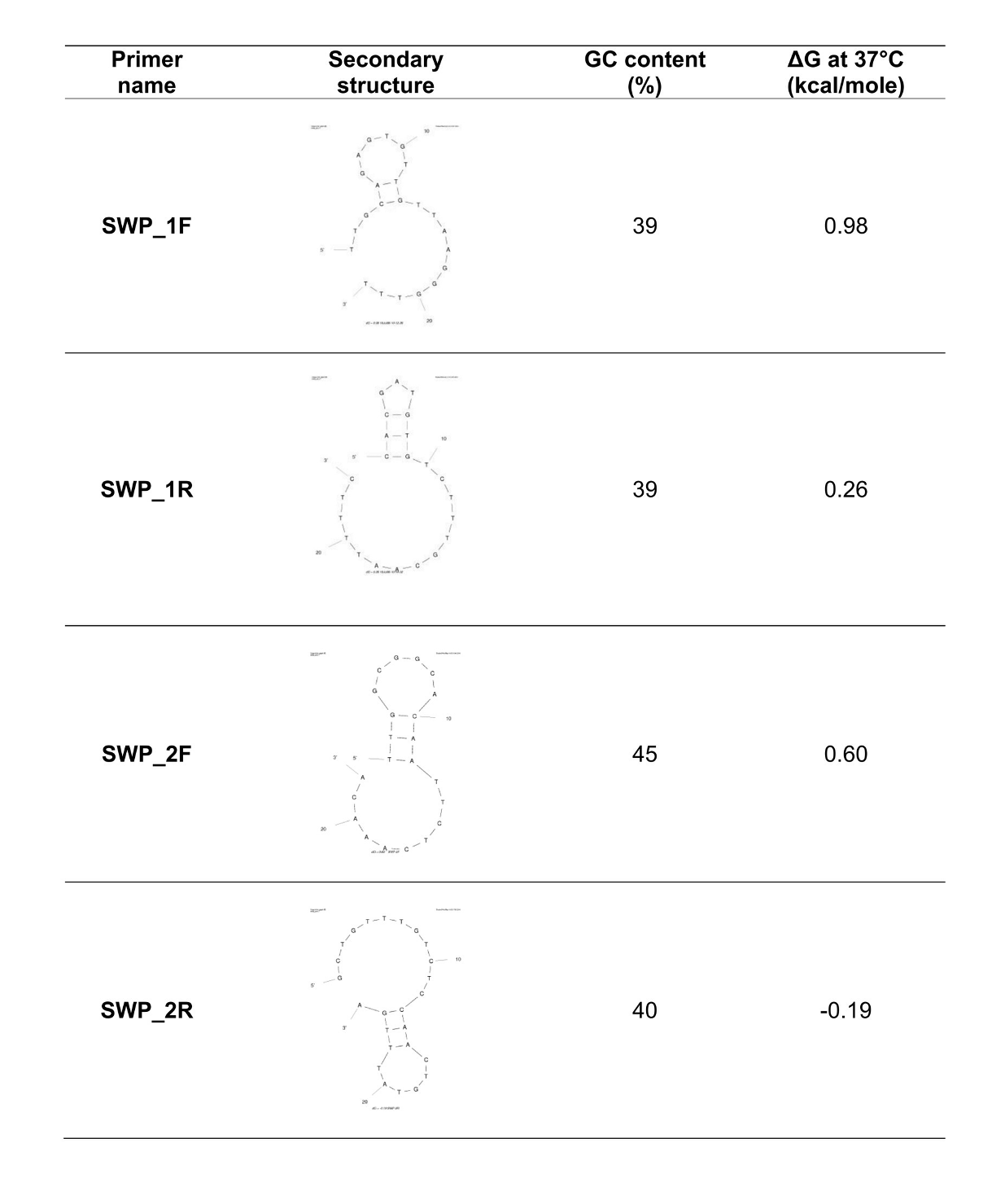
**

Supplement: S1 Table — (DOCX) [file pone.0166320.s001.docx]

**S2 Table. GC content and stability of secondary structures of the SSU rRNA primers.**


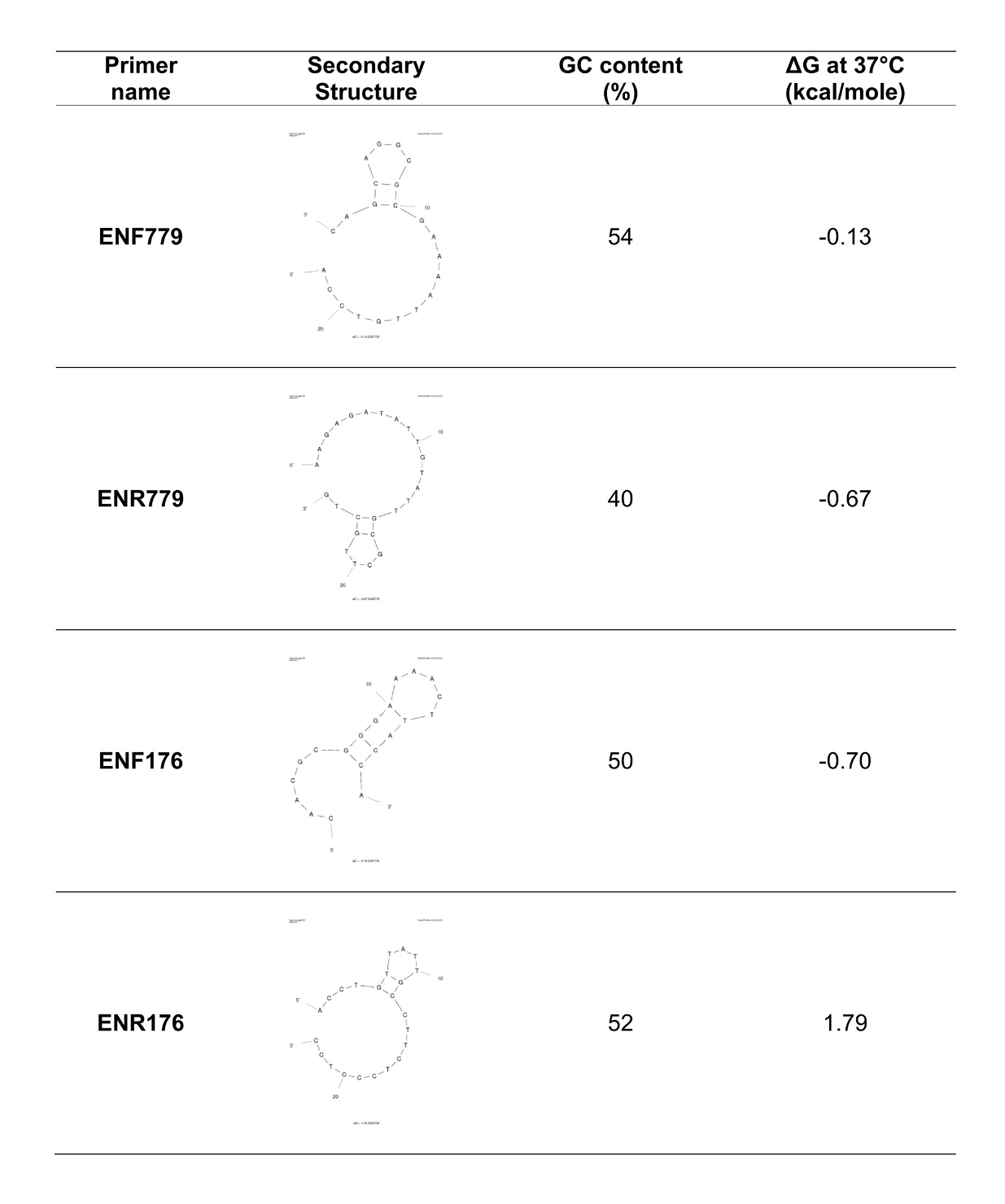

Supplement: S2 Table — (DOCX) [file pone.0166320.s002.docx]
